# Splinting of penis after micro vascular reconstruction: A simple inexpensive method

**DOI:** 10.4103/0970-0358.63945

**Published:** 2010

**Authors:** Nikhil Panse, Parag Sahasrabudhe, Sanjay Date, Sachin Balwantkar

**Affiliations:** Department of Plastic Surgery, B. J. Medical College & Sasoon Hospital, Pune, India

Sir

We would like to congratulate the authors.[1] for coming up with an innovative idea for splintage of the penis after penile reconstruction. The authors have used two thermocol cups, cut from the bases and stuck together.[1] This forms a near ideal dressing for penile reconstruction where there is no compression; one can monitor the flap and is cheap too. However, we would like to share our views regarding penile splintage.

**Figure 1:**
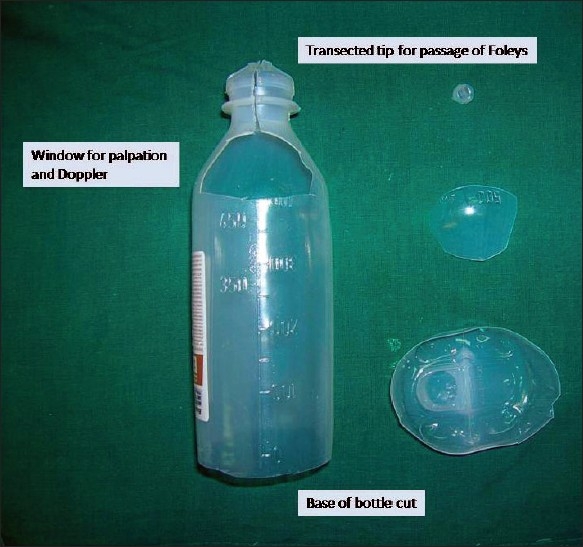
Making of the splint

**Figure 2:**
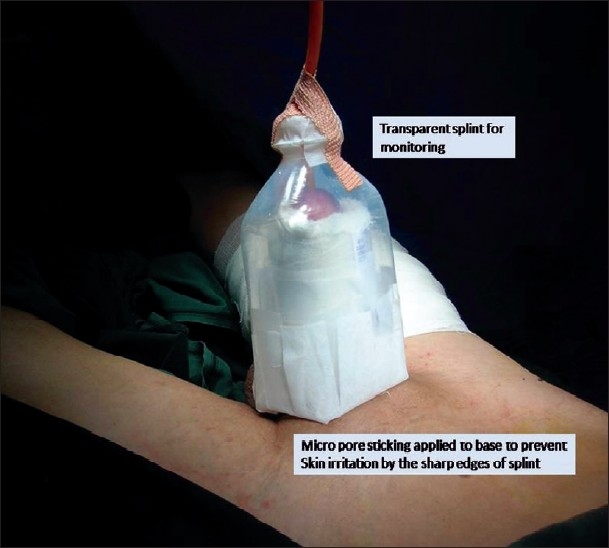
Splint application

**Figure 3:**
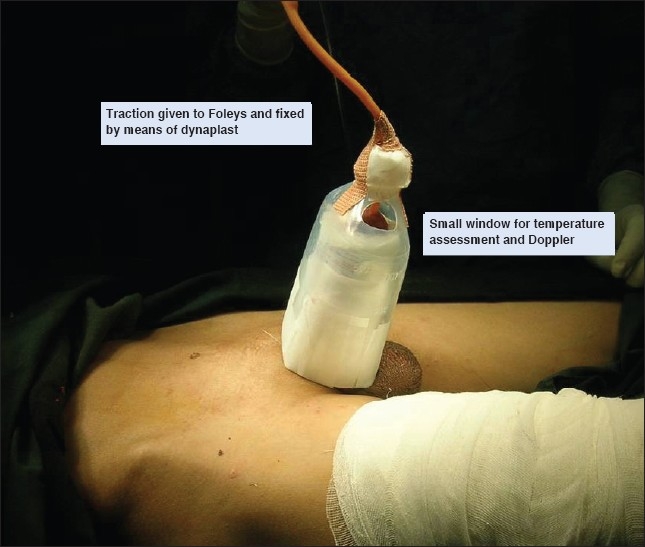
Splint showing window for palpation and/or doppler

We use a used plastic saline bottle for this purpose. It is cut at the base and near its neck and slided over the penis. The Foleys is removed through the tip of the saline bottle and it is fixed to the bottle by an adhesive tape. The sharp edges of the base of the saline bottle are covered with micropore or white sticking to prevent irritating the patient's abdominal wall skin. This particular splint has all the advantages needed for a penile dressing [Figures Figures 1‐3].

1. There is no compression over the penis, and loose dressings can be given.
2. Since the saline bottle is transparent, one can easily monitor the flap and dressing for soakage, etc.
3. Through the window over the top, one can palpate and if necessary Doppler the penis in case for penile replantations and reconstructions.
4. Traction if needed can be given over the Foleys catheter, and then fixed to the saline bottle. Thus it is also helpful in case of skin grafting where traction would be beneficial. In case of reconstruction, the traction helps to keep the penis from buckling and folding on itself and adds to the venous drainage.
5. The splint is very light weight and it can be taped to the abdominal wall for stability.
6. This splint is made of used plastic saline bottles (waste) and can be discarded at will. It is simple to make the splint, apply it and remove it. Flap monitoring is easy, there is no discomfort to the patient and it can be used for grafting as well as reconstructions.

To conclude, our splint has all the advantages of the various dressing materials described in literature for penile splintage without any significant disadvantages.
